# Is the Greulich and Pyle atlas applicable to all ethnicities? A systematic review and meta-analysis

**DOI:** 10.1007/s00330-018-5792-5

**Published:** 2019-01-07

**Authors:** Khalaf Alshamrani, Fabrizio Messina, Amaka C. Offiah

**Affiliations:** 1 Department of Oncology & Metabolism, University of Sheffield, Sheffield, UK; 2 College of Applied Medical Sciences, Najran University, Najran, Saudi Arabia; 3 Academic Unit of Child Health, Damer Street Building, Sheffield Children’s NHS Foundation Trust, Western Bank, Sheffield, S10 2TH UK; 4 School of Health and Related Research, University of Sheffield, Sheffield, UK; 5 Leeds Institute of Clinical Trials Research, University of Leeds, Leeds, UK

**Keywords:** Age determination by skeleton, Forensic medicine, X-rays, Meta-analysis

## Abstract

**Objective:**

To determine whether the Greulich and Pyle (G&P) atlas is applicable when applied to populations of different ethnicity.

**Methods:**

A systematic review of studies published between 1959 and 15th February 2017 identified from the Embase, MEDLINE and Cochrane databases was undertaken. Quality of the studies was assessed using the National Institute for Health and Care Excellence tool. Meta-analysis used mean differences and standard deviations as summary statistics for the difference between bone age (BA) and chronological age (CA).

**Results:**

A total of 49 studies were included of which 27 (55%) were related to Caucasian populations. Of the 49 eligible studies, 35 were appropriate for further meta-analysis. In African females, meta-analysis showed a significant mean difference between BA and CA of 0.37 years (95% CI 0.04, 0.69). In Asian males, meta-analysis showed significant differences between BA and CA of -1.08, -1.35, -1.07, -0.80 and 0.50 years for chronological ages of 6, 7, 8, 9 and 17 years, respectively. Meta-analysis showed no significant differences between BA and CA in African males, Asian females, Caucasians and Hispanics.

**Conclusions:**

The G&P standard is imprecise and should be used with caution when applied to Asian male and African female populations, particularly when aiming to determine chronological age for forensic/legal purposes.

**Key Points:**

*• In African females, bone age is significantly advanced when compared to the G&P standard.*

*• In Asian males, bone age is significantly delayed between 6 and 9 years old inclusive and significantly advanced at 17 years old when compared to the G&P standard.*

*• The G&P atlas should be used with caution when applied to Asian and African populations, particularly when aiming to determine chronological age for forensic/legal purposes.*

**Electronic supplementary material:**

The online version of this article (10.1007/s00330-018-5792-5) contains supplementary material, which is available to authorized users.

## Introduction

Determining maturity and understanding growth in a child is critical for medical and psychosocial purposes. Assessing bone age is important to investigate whether the maturity of bones is occurring at the same rate as the chronological ageing process. Furthermore, bone age assessment has a role in forensic and legal investigations when the individual’s chronological age is in doubt. For example, in asylum seekers and unaccompanied minors without valid documents to prove their ages [1], it is important to assess bone age using a reliable and suitable method [2]. Incorrectly assessing a child as an adult leaves the child with limited access to education, healthcare and other support provided to children.

There are two approaches widely used to determine bone age from a left hand radiograph: the Greulich and Pyle (G&P) and Tanner and Whitehouse (TW) methods [3, 4]. The population which formed the G&P standard atlas were North American Caucasians of good socioeconomic status. The assessment process is typically based on comparing a hand-wrist radiograph of a child with the age-matched standard radiographs as contained in the atlas. The G&P method depends on comparing the overall maturational status and is known to be straightforward and quick, therefore widely used. In contrast, the TW method depends on assessing and scoring the skeletal maturity of each individual bone of the hand, hence taking a longer time than the G&P method. Since the establishment of the G&P atlas, many studies have been conducted in different parts of the world to determine whether it is applicable to different populations. This question is important, particularly given the increasing legal and illegal influx of immigrants to certain parts of Europe. This systematic review and meta-analysis aims to provide a better understanding of the applicability of the G&P atlas to children and adolescents who are of a different population from the original standard.

## Materials and methods

### Search strategy

A systematic search of the MEDLINE, Embase and Cochrane databases was conducted. We searched MEDLINE using keywords ((Greulich and Pyle)) OR Greulich Pyle, ((bone age assessment OR bone age determination)) AND left hand and refined the search to include articles in English published between 1st January 1959 and 15th February 2017. No free text was used in this search. For Embase, we used the term (Greulich and Pyle) and refined the search to include articles in English published between 1st January 1959 and 15th February 2017. We also searched the Cochrane library using the keywords (Greulich and Pyle) and the MeSH term (Age Determination by Skeleton). The search was refined to include articles in English published between 1st January 1959 and 15th February 2017. Each study’s title and abstract was screened to determine whether it presented data correlating bone age assessed by the G&P with chronological age. The full text was retrieved when the reviewers could not decide on the study’s eligibility from the title and abstract alone. The following exclusion criteria were then applied:

1. Health status of participants could not be confirmed from the article or participants with developmental disorders or subjected to nutritional supplementation (these represent unhealthy children expected to show delayed or advanced bone age).
2. Using a modified method of G&P and/or using modalities other than conventional radiography
3. Full text not available within the resources available to the reviewers
4. Full text not in English
5. Review articles
6. When the mean difference between bone age (BA) and chronological age (CA) was not reported or could not be calculated by the reviewers based on the study results presented.

The search was independently carried out by two reviewers (KA and ACO), followed by a consensus meeting to agree the final selection of studies for inclusion in this review.

### Quality assessment

Two reviewers KA and ACO independently assessed the quality of included studies using the tool developed by the National Institute for Health and Care Excellence (NICE, Appendix G) [5]. Discrepancies were resolved by discussion. The tool considers five aspects of a study: population, method of participant selection, outcomes, analysis and generalisability of the study. Then, an overall study quality grading is given to each study for internal validity (IV) and a separate grading for external validity (EV) as follows:

- ++ All/most of the checklist criteria have been fulfilled and the conclusions are unlikely to alter.
- + Some of the checklist criteria have been fulfilled; the conclusions are unlikely to alter even when they have not been fulfilled.
− Few or no checklist criteria have been fulfilled, and the conclusions are likely or very likely to alter.

### Data extraction

A single reviewer (K.A.) extracted and recorded the following data from eligible studies:

1. Sample size (males and females)
2. Ethnicity or country of origin
3. Mean difference and standard deviation (SD) between bone age and chronological age (BA-CA)
4. Mean and SD of bone age
5. Mean and SD of chronological age
6. Authors’ conclusions
7. Applicability of the standard

Given the review question, studies were divided into four groups based on major ethnic groups: African, Asian, Caucasian and Hispanic. Data for each major ethnic group were summarised and analysed separately. Some studies reported the place/country from which participants were recruited, and in such cases, the study was grouped under the major ethnicity of that country. The mean differences between BA and CA are to be interpreted as follows: a positive value indicates that the child’s bone age exceeds the child’s chronological age and a negative value indicates delayed bone age compared to chronological age.

Additionally, we defined four categories to reflect the applicability of the G&P standard to the studied population as follows: (a) applicable, (b) not applicable (determined by the authors’ use of words identical or similar to “applicable” or “not applicable”, respectively, in the study’s discussion or conclusion), (c) needs some modification (authors use phrases such as, “can be used with caution” or when the standard was found to be applicable to a certain age group but not others) and (d) not clear (when the study failed to mention whether the standard was applicable, not applicable or needed modification).

### Statistical analysis

A combination of random effect meta-analyses by ethnicity (African, Asian, Caucasian and Hispanic) and sex was conducted using R Software [6]. Overall meta-analysis of all ethnicities was also determined. Additionally, meta-regression with covariates analysis (including sex and ethnicity as explanatory variables) was determined. Yearly interval sub-analysis of Asians aged 6 to 17 years and Caucasians aged 10 to 17 years was carried out in males and females. Other ethnicities were excluded from interval sub-analysis as the age groups were not constant between studies.

In total, 50 meta-analyses were performed using mean differences and standard deviations as summary statistics for the difference between bone age and chronological age. When a study examined more than one ethnicity, each ethnicity was treated as a separate study (only for the meta-analysis). Heterogeneity was assessed between 0 (no heterogeneity) and 100% (maximum heterogeneity) using the *I*-squared statistic. A funnel plot was determined to assess bias or the present of any systematic heterogeneity.

## Results

**Fig. 1:**
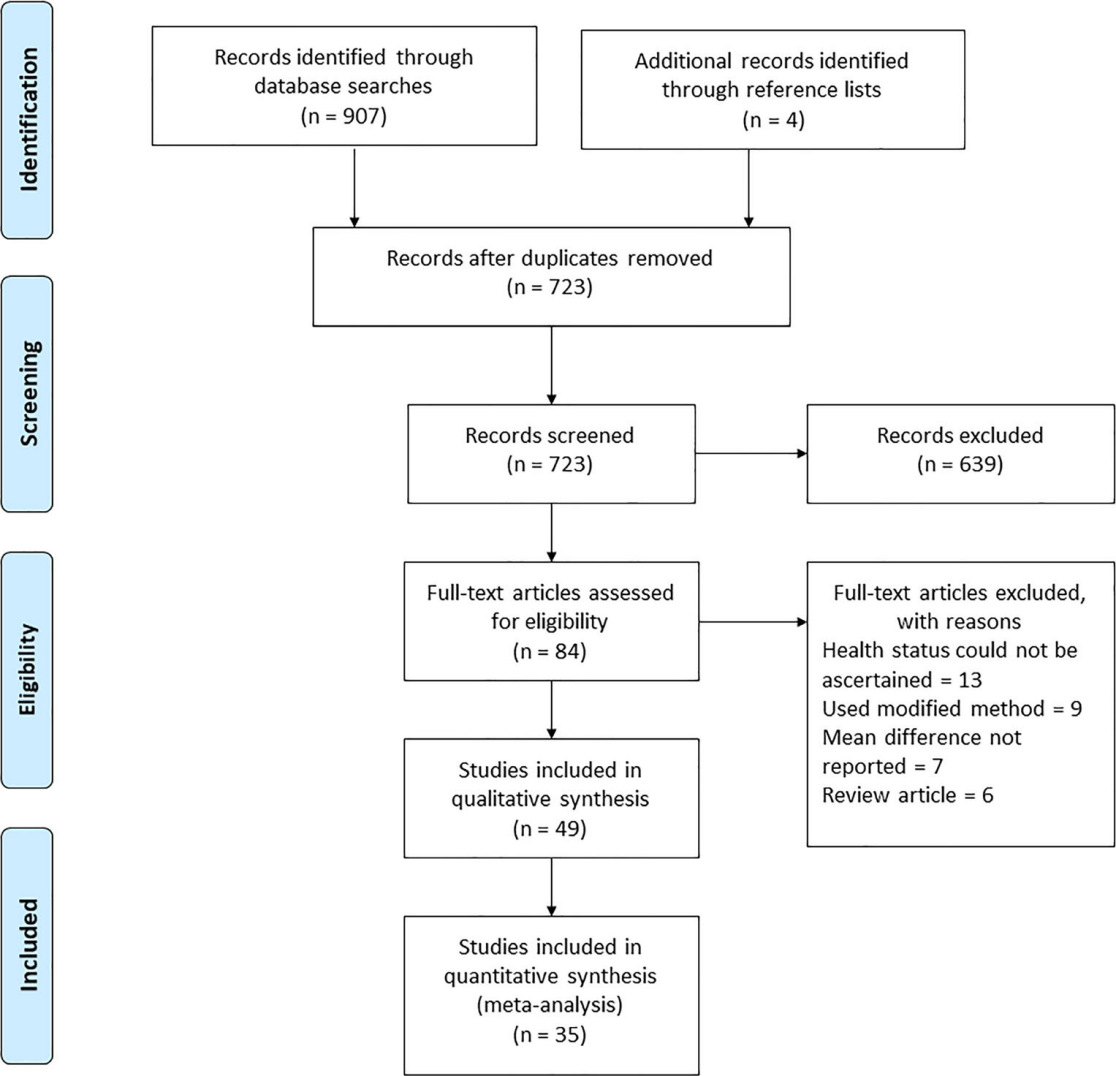
Flow chart to show article selection process

This systematic review identified 907 studies of which 45 were eligible for inclusion (Fig. 1). Four additional studies were identified from the reference lists of the initial 45 extracted papers; therefore, the total number of included studies was 49 [7–55], of which 27 (55%) were related to Caucasian populations. The total number of children in the included studies was 21,081 (11,445 boys), comprising 11,194 Caucasians (5922 boys), 6776 Asians (3731 boys), 1705 Africans (1073 boys) and 1406 Hispanics (781 boys). As summarised in Table 1, there was minimal risk of bias for internal validity alone in one study [33], for external validity alone in five studies [17, 18, 25, 40, 50] and for both internal and external validity in 12 studies [11, 20–22, 27, 30, 35, 42, 43, 45, 51, 54]. There was significant risk of bias for internal validity alone in 0 studies, for external validity alone in two studies [8, 46] and for both internal and external validity in 2 studies [23, 29]. Sources of bias in these four studies requiring that their results be interpreted with caution include:

1. Absent documentation of statistical criteria such as *p* values and/or observer reliability [8, 23]
2. Insufficient detail about the source of the study population [29]
3. Non-representative samples [46]

**Table 1:** Quality assessment of the included studies (after agreement between the two assessors)

Study [Reference]	IV*	EV**	Study	IV*	EV**
Demish and Wartmann 1956 [7]	+	+	Calfee et al 2010 [32]	+	+
Hansman and Maresh 1961 [8]	+	-	Zafar et al 2010 [33]	++	+
Johnston 1963 [9]	+	+	Santos et al 2011 [34]	+	+
Andersen 1971 [10]	+	+	Cantekin et al 2012 [35]	++	++
Roche et al 1971 [11]	++	++	Dembetembe and Morris 2012 [36]	+	+
Wenzel et al 1984 [12]	+	+	Moradi et al 2012 [37]	+	+
So and Yen 1990 [13]	+	+	Patil et al 2012 [38]	+	+
So 1991 [14]	+	+	Santoro et al 2012 [39]	+	+
Loder et al 1993 [15]	+	+	Soudack et al 2012 [40]	+	++
Kullman 1995 [16]	+	+	Suri et al 2013 [41]	+	+
Ontell et al 1996 [17]	+	++	Hackman and Black 2013 [42]	++	++
Jiménez-Castellanos et al 1996 [18]	+	++	Paxton et al 2013 [43]	++	++
Koc et al 2001 [19]	+	+	Shilpa et al 2013 [44]	+	+
Mora et al 2001 [20]	++	++	Awais et al 2014 [45]	++	++
Van Rijn et al 2001 [21]	++	++	Rai et al 2014 [46]	+	-
Krailassiri et al 2002 [22]	++	++	Mansourvar et al 2014 [47]	+	+
Lewis et al 2002 [23]	-	-	Mughal et al 2014 [48]	+	+
Chiang et al 2005 [24]	+	+	Gungor et al 2015 [49]	+	+
Garamendi et al 2005 [25]	+	++	Kim et al 2015 [50]	+	++
Haiter-Neto et al 2006 [26]	+	+	Mohammed et al 2015 [51]	++	++
Büken et al 2007 [27]	++	++	Öztürk et al 2015 [52]	+	+
Griffith et al 2007 [28]	+	+	Patel et al 2015 [53]	+	+
Schmidt et al 2007 [29]	-	-	Zabet et al 2014 [54]	++	++
Büken et al 2009 [30]	++	++	Maggio et al 2016 [55]	+	+
Zhang et al 2009 [31]	+	+			

*IV: internal validity, **EV: external validity

++ Indicates that the study has been designed or conducted in such a way as to minimise the risk of bias

+ Indicates that either the answer to the checklist question is not clear from the way the study is reported, or that the study may not have addressed all potential sources of bias

- Reserved for those aspects of the study design in which significant sources of bias may persist

Studies included in this systematic review reported the mean difference between bone age and chronological age in different forms. Twenty-nine studies (60%) [8–14, 18, 19, 22, 24, 26–28, 30, 35, 36, 38, 41, 42, 44, 45, 48–54] presented the mean difference for each year of age for each sex. In such cases, the maximum delay and advancement in bone age was extracted. Twelve studies [15, 17, 20, 25, 31, 33, 34, 37, 39, 40, 46, 47] divided their sample into subgroups, where each subgroup contains up to five age groups, e.g. children aged between 1 and 5 years old. For each subgroup, the overall mean difference for each sex is reported. Eight studies [7, 16, 21, 23, 29, 32, 43, 55] only reported the overall mean difference between bone age and chronological age, limiting the applicability of their results to individual age groups. Data relating to ethnicity or country of origin, sample size, mean BA-CA and the authors’ conclusions are summarised for each study in Tables 2, 3, 4, and 5.

**Table 2:** Summary of studies that assessed the reliability of the G&P atlas in Caucasian children

Study	Origin/ethnicity	Age (years)	*N*	Mean BA-CA (years)	Authors’ conclusion	Applicability
Demish and Wartmann 1956	White	9–15	M = 81 F = 70	M = 0 F = 0.5	There is a high positive correlation between BA and CA.	Applicable
Hansman and Maresh 1961	White	0–18	M = 27 F = 36	M = -0.33 F = 0.75	The mean BA for both sexes is equal to CA during infancy but less than CA toward adolescents.	Applicable
Johnston 1963	White	7–17	M = 388 F = 405	M = 0.40 F = 0.20	Children show significant differences between CA and BA.	Needs some modification
Andersen, 1971	Danish	7–18	M = 477 F = 535	M = 0.49 F = -0.43	BA is lower than CA, indicating that the American children mature earlier than the Danish.	Needs some modification
Roche et al 1971	British	2–13	M = 62 F = 82	M = 0.01 F = 0.07	Children matured skeletally at about the same as the G&P standard.	Applicable
Wenzel et al 1984	Austrian	7–16	M = 459 F = 178	M = -0.2 F = -0.13	Major deviations between BA and CA were at and after puberty.	Not clear
Loder et al 1993	White	0–18	M = 203 F = 177	M = -0.1 F = 0.07	The G&P atlas is applicable to white girls at all ages and white boys in early childhood (less than 4 years old). BA of white boys was delayed during middle and late childhood but advanced during adolescence by 5 years.	Not applicable
Kullman, 1995	Swedish	12–19	M = 38 F = 34	M = -0.4 F = -0.4	It is recommended to assess skeletal development using G&P.	Applicable
Ontell et al 1996	White	3–18	M = 208 F = 130	M = -0.29 F = 0.14	The G&P standard is applicable to white girls at all ages, while in boys it can only be applied in adolescence.	Applicable (for girls but not boys)
Koc et al 2001	Southeast Turkey	7–17	M = 225	M = -0.2	Mean BA was delayed between 7 and 13 years old and then advanced between 14 and 17 years. The atlas can be used with some modification.	Needs some modification
Mora et al 2001	European American	0–19	M = 130 F = 130	M = 0.09 F = -0.14	Prepubertal European American children have significantly delayed BA when compared to African American children. Post-pubertal European-American males have significantly advanced BA when compared with African American males. A new standard is needed for reliable BA assessment.	Not applicable
Van Rijn et al 2001	Dutch	5–20	M = 294 F = 294	M = -0.28 F = -0.14	Significant correlation between BA and CA in boys and girls. The G&P atlas is still applicable to Dutch Caucasian children and adolescents.	Applicable
Buken et al 2007	Turkish	11–19	M = 251 F = 241	M = 0.13 F = 0.54	Mean skeletal ages were significantly advanced for boys and girls between 11 and 17 years old. The cause of this acceleration might be new social and cultural factors rather than economic conditions.	Needs some modification
Schmidt et al 2007	Germany	1–18	M = 303 F = 303	M = -0.49 F = -0.39	The G&P atlas method overestimated the samples’ age. This may be due to high acceleration in growth.	Applicable
Buken et al 2009	Turkish	11–16	M = 169 F = 164	M = -0.02 F = -0.65	The G&P atlas is appropriate in girls 11–15 years old and boys 11–16 years old from the Black Sea region of Turkey.	Needs some modification
Zhang et al 2009	White	0–18	M = 164 F = 163	M = 0.01 F = -0.15	BA was relatively close to CA in white children.	Applicable
Calfee et al 2010	Caucasian	12–18	M = 62 F = 76	M = 0.98 F = 0.66	American children between 12 and 18 years demonstrate BA exceeding CA. Females between 12 and 15 years old are most likely to demonstrate a discrepancy of at least 2 years between BA and CA, while males demonstrate this throughout adolescence.	Not clear
Cantekin et al 2012	Eastern Turkish	7–17	M = 342 F = 425	M = -0.13 F = 0.20	The mean differences between BA and CA are low enough to be of no practical significance, and thus, this method can be used in all age groups within the current study.	Needs some modification
Santoro et al 2012	Italian	7–15	M = 243 F = 261	M = -0.1 F = 0.40	The G&P method is accurate, particularly in the age ranges of 7–9 years and 10.4–11.5 years.	Applicable
Suri et al 2012	White	9–18	M = 311 F = 261	M = 0.50 F = 0.50	Wide range of differences between BA and CA at each yearly age group from 9 to 18 years. Overall, the differences in skeletal and chronological age were positively correlated.	Not clear
Hackman and Black 2013	Scottish	1–20	M = 249 F = 157	M = -0.13 F = -0.16	The G&P atlas over-aged females from birth until 13 years of age and underestimated males from birth until 13 years of age after which point it consistently over-aged boys between 13 and 17 years of age.	Needs some modification
Paxton et al 2013	Australian	0–18	M = 276 F = 130	M = -0.12 F = -0.30	The G&P atlas is an accurate means of BA determination in Australian children.	Applicable
Mansourvar et al 2014	White	10–16	M = 46	M = 0.04	The G&P is reliable in Caucasian males.	Applicable
Gungor et al 2015	Turkish	10–18	M = 259 F = 276	M = 0.64 F = -0.98	It is appropriate to use the G&P method in southern Turkish children; however, a revision is needed for better results and to minimise errors.	Needs some modification
Zabate et al 2015	French	10–19	M = 100 F = 90	M = -0.19 F = -0.53	The G&P overestimated all males and females except boys who are 12 years and girls who are 11 and 18 years old. G&P can be used on French population but not without caution because of a tendency for this method to overestimate age.	Needs some modification
Ozturk et al 2015	Central Turkey	9–17	M = 186 F = 249	M = -0.10 F = 0.90	The G&P atlas was applicable to Caucasian boys of younger age groups and Caucasian girls of all ages. However, some improvement is needed.	Needs some modification
	Eastern Turkey	9–17	M = 189 F = 225	M = -0.90 F = -0.90		
Maggio et al 2016	Western Australian	0–25	M = 180 F = 180	M = 0.24 F = -0.14	The G&P standard is not suitable for the determination of legal majority.	Not clear

A positive value of the mean difference between BA and CA indicates advanced, while a negative value indicates delayed bone age compared to chronological age

*M* males, *F* females

**Table 3:** Summary of studies that assessed the reliability of the G&P atlas in African children

Study	Origin/ethnicity	Age (years)	*N*	Mean BA-CA (years)	Authors’ conclusion	Applicability
Loder et al 1993	African American	0–18	M = 249 F = 212	M = 0.28 F = 0.51	African girls were skeletally advanced by 0.4 to 0.7 years except during middle childhood. While BA for boys was only advanced during adolescence.	Applicable for boys but not for girls
Ontell et al 1996	African American	3–18	M = 95 F = 65	M = 0.28 F = 0.55	African girls showed significant differences at all ages except middle childhood. G&P is applicable to African boys until adolescence.	Applicable for boys but not for girls
Mora et al 2001	African American	0–19	M = 135 F = 139	M = -0.01 F = 0.11	On average, the BA of 10% of prepubertal African American children was 2 SD above the normative data in the G&P atlas. The atlas is imprecise for African American children born after 1980.	Not applicable
Lewis et al 2002	Malawian	1–28	M = 93 F = 46	M = -1.7 F = -1.5	The atlas is inaccurate for this group of children. Poor nutrition and chronic diseases such as malaria and diarrhoea which are endemic in Malawi are likely to be contributing factors**.**	Not applicable
Garamendi et al 2005	Moroccan	13–25	M = 144	M = -1.7	G&P has a high error rate and therefore should not be considered as an optimal diagnostic method.	Not applicable
Zhang et al 2009	African American	0–18	M = 179 F = 170	M = -0.02 F = 0.03	BA was relatively close to the CA in African American children.	Applicable
Dembetembe and Morris 2012	South African (black)	13–19 20–21	M = 104 M = 27	M = 0.2 M = 2.1	Skeletal maturity as characterised by complete epiphyseal fusion occurred approximately 2.1 years later than G&P method. G&P is not directly applicable to African males.	Not applicable
Mansourvar et al 2014	African American	8–15	M = 47	M = 1.87	G&P is not reliable for assessment of children between 8 and 15 years.	Needs some modification

A positive value of the mean difference between BA and CA indicates advanced, while a negative value indicates delayed bone age compared to chronological age

*M* males, *F* females

**Table 4:** Summary of studies that assessed the reliability of the G&P atlas in Asian children

Study	Origin/ethnicity	Age (years)	*N*	Mean BA-CA (years)	Authors’ conclusion	Applicability
So and Yen 1990	Southern Chinese	11.9–12.3	F = 117	F = 0.6	Earlier skeletal maturation was demonstrated. Such a difference was contributed to by improved socioeconomic, nutritional and sociohygienic conditions.	Not clear
So and Yen 1991	Southern Chinese	11.9–12.3	F = 117	F = 0.6	Earlier skeletal maturation was demonstrated. This is attributed to improved socioeconomic condition.	Not clear
Ontell et al 1996	Asian	3–18	M = 63 F = 30	M = -0.03 F = 0.27	The G&P standard is applicable to Asian girls at all ages, while in boys, it can only be applied from birth to 4 years old and from 7 to 13.3 years old.	Applicable (for girls but not boys.)
Krailassiri et al 2002	Thai	7–19	M = 139 F = 222	M = 0.8 F = 0.8	Although the mean difference in BA and CA was equal in both sexes, males clearly differed from the G&P more frequently than females.	Not applicable
Chiang et al 2005	Taiwan	7–19	M = 230 F = 140	M = 0.82 F = -0.3	There is a discrepancy of more than 1 year between BA and CA in some age groups. We believe that some modification of the GP atlas is necessary	Needs some modification
Griffith et al 2007	Chinese	0–18	M = 650 F = 366	M = 0.25 F = 0.15	Hong Kong children appear to mature more slowly in the first decade but more quickly thereafter.	Needs some modification
Zhang et al 2009	Asian	0–18	M = 165 F = 166	M = 0.41 F = 0.24	Asian children mature sooner than white children, especially between 10 and 13 years in girls and between 11 and 15 years in boys.	Not clear
Zafer et al 2010	Pakistan	0–18	M = 535 F = 354	M = 0.1 F = -0.19	This study suggests against the applicability of G&P in Pakistani children. Authors propose a cautious approach while employing G&P in this population to ensure appropriate clinical and medico-legal decisions.	Not applicable
Moradi et al 2012	Iran	6–18	M = 303 F = 122	M = 0.37 F = -0.04	Considering the possibility of a few months’ difference, the G&P atlas can be used for the Iranian population.	Needs some modification
Soudack et al 2012	Israeli	0–18	M = 375 F = 304	M = 0.16 F = -0.04	There was no discrepancy between BA and CA in Israeli girls using G&P. There were discrepancies for boys, but these were small.	Applicable
Patil et al 2012	India	1–19	M = 194 F = 181	M = 0.69 F = 0.64	G&P is not applicable to males, especially for age group 4 to 12 years. G&P is applicable to females except age groups 4–7 years, 9–10 years, 15–16 years. A new standard is needed for Indian children.	Not applicable
Shilpa et al 2013	India (Bangalore)	6–15	M = 124 F = 126	M = 0.18 F = 0.29	The G&P method of skeletal age estimation showed accuracy in only certain age groups in Bangalore South zone children.	Needs some modification
Awais et al 2014	Pakistani	0–18	M = 136 F = 147	M = -1.3 F = 0.06	G&P is reliable for girls in all age groups. However, G&P is not accurate for boys in whom it underestimated BA.	Not applicable
Mansourvar et al 2014	Asian American	1–8	M = 48	M = 0.87	The delay in skeletal maturity was more than 2 years for the 4–6 years’ age group. Some improvement is needed to enhance the precision of G&P.	Needs some modification
Mughal et al 2014	Pakistan	4.5–9.5	M = 139 F = 81	M = -1.3 F = 0.55	G&P standard significantly underestimates CA in Pakistani children between the ages of 4.5 and 9.5 years.	Not applicable
Rai et al 2014	India	5–15	M = 75 F = 75	M = -0.07 F = -0.33	G&P atlas underestimates CA in children aged between 5 and 9 years.	Needs some modification
Kim et al 2015	Korean	7–12	M = 135 F = 77	M = -0.48 F = -0.02	G&P is applicable to Korean children aged between 7 and 12 years.	Applicable
Mohammed et al 2015	South India	9–20	M = 330 F = 330	M = -0.23 F = 0.02	Mild underestimation of BA was noted in boys. G&P remains applicable to South Indian children.	Needs some modification
Patel et al 2015	West India	6–16	M = 90 F = 90	M = -0.99 F = -0.40	G&P can be used in West Indian children aged between 6 and 16 years.	Applicable

A positive value of the mean difference between BA and CA indicates advanced, while a negative value indicates delayed bone age compared to chronological age

*M* males, *F* females

**Table 5:** Summary of studies that assessed the reliability of the G&P atlas in Hispanic children

Study	Origin/ethnicity	Age (years)	*N*	Mean Ba-CA (years)	Authors’ conclusion	Applicability
Jimenez et al 1996	Spanish	0–14	M = 139 F = 100	M = -0.31 F = 0.04	Boys show a delay of around 3 months with respect to the G&P atlas. Girls show a better fit to the corresponding (female) standard of the atlas.	Not clear
Ontell et al 1996	Hispanic	3–18	M = 105 F = 69	M = 0.28 F = 0.38	The G&P atlas is applicable to boys aged between 4 and 13 years and to girls except during adolescence.	Needs some modification
Haiter-Neto et al 2006	Brazilian	7–15	M = 180 F = 180	M = -0.2 F = 0.1	The means of estimated and chronologic ages were similar in all age ranges. The standards can be used with some modification.	Needs some modification
Zhang et al 2009	Hispanic	0–18	M = 178 F = 182	M = 0.30* F = 0.24*	Hispanic children mature sooner than the G&P atlas, especially between 10 and 13 years of age in girls and between 11 and 15 years of age in boys.	Not clear
Santos et al 2011	Portuguese	12–20	M = 136 F = 94	M = 0.12 F = 0.02	The G&P atlas can be used; however, caution must be taken at the end of the growing period.	Needs some modification
Mansourvar et al 2014	Hispanic	15–18	M = 43	M = 0.37	The G&P method is reliable in Hispanic males.	Applicable

A positive value of the mean difference between BA and CA indicates advanced, while a negative value indicates delayed bone age compared to chronological age

*M* males, *F* females

*p < 0.05

### Meta-analysis based on ethnicity

**Fig. 2:**
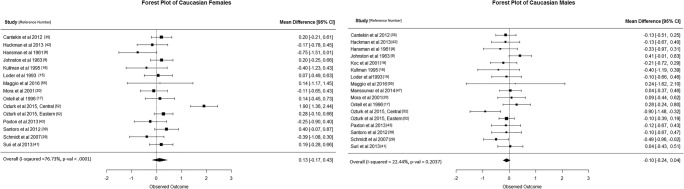
Forest plot of Caucasians (females and males)

**Fig. 3:**
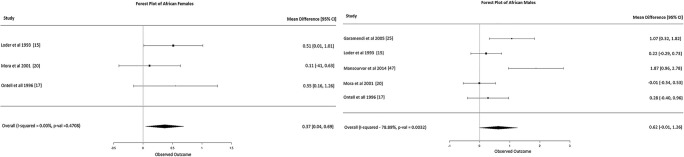
Forest plot of Africans (females and males)

**Fig. 4:**
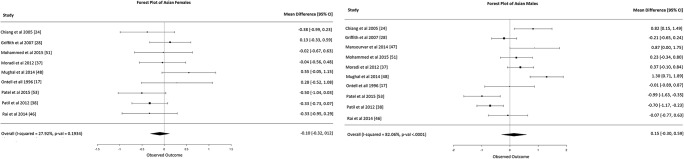
Forest plot of Asians (females and males)

**Fig. 5:**
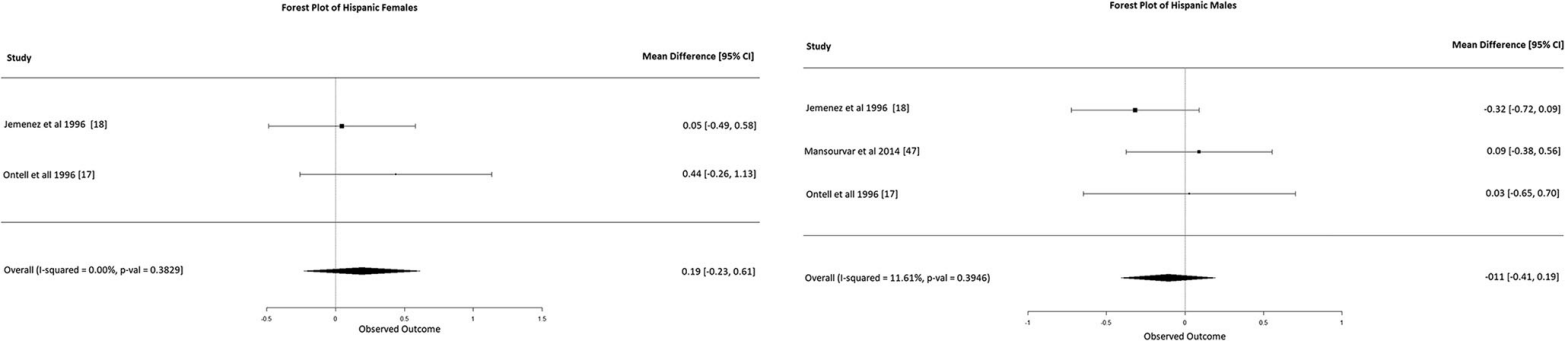
Forest plot of Hispanics (females and males)

1. Caucasian females: Fifteen studies were included in the meta-analysis. These 15 studies presented moderate heterogeneity (*I*-squared 76%, Fig. 2) but did not show any statistically significant results, with overall mean difference BA-CA of 0.13 years (95% CI -0.17, 0.43).
2. Caucasian males: Seventeen studies were included in the meta-analysis. These 17 studies presented low heterogeneity (*I*-squared 22%, Fig. 2) and did not show any statistically significant results, with an overall mean difference BA-CA of -0.10 years (95% CI, -0.24, 0.04).
3. African females: Only three studies were included in the meta-analysis. The three studies were homogeneous (*I*-squared 0%, Fig. 3) and showed statistically significant results, with overall mean difference BA-CA of 0.37 years (95% CI 0.04, 0.69).
4. African males: Only five studies were included in the meta-analysis. The five studies presented moderate heterogeneity (*I*-squared 78%, Fig. 3) but did not show any statistically significant results, with overall mean difference BA-CA of 0.62 years (95% CI -0.01, 1.26).
5. Asian females: Only nine studies were included in the meta-analysis. These nine studies presented low to moderate heterogeneity (*I*-squared 27%, Fig. 4) but did not show any statistically significant results, with overall mean difference BA-CA of -0.10 years (95% CI -0.32, 0.12).
6. Asian males: Ten studies were included in the meta-analysis. The studies were highly heterogeneous (*I*-squared 82%, Fig. 4) but did not show any statistically significant results, with overall mean difference BA-CA of 0.15 years (95% CI -0.30, 0.59).
7. Hispanic females: Only two studies were included in the meta-analysis. The two studies presented no heterogeneity (*I*-squared 0%, Fig. 5) and did not show any statistically significant results, with overall mean difference BA-CA of 0.19 years (95% CI -0.23, 0.61).
8. Hispanic males: Only three studies were included in the meta-analysis. The three studies presented low heterogeneity (I-squared 11%, Fig. 5) but did not show any statistically significant results, with overall mean difference BA-CA of -0.11 years (95% CI -0.41, 0.19).

In regard to the meta-regression, the coefficient for the Africans showed statistical significance with estimate being (*p* > 0.05) (Supplementary Table 2).

### Meta-analyses by yearly interval (see also Supplementary Tables 3 to 6 and Supplementary Figs. 1 to 5)

For Caucasian males, seven studies were included [9, 19, 27, 30, 35, 41, 52]. These studies did not show any statistically significant results. The mean difference BA-CA ranged from -0.32 years (at 13 years old) to 0.44 years (at 17 years old). For Caucasian females, six studies were included [9, 27, 30, 35, 41, 52]. These studies did not show any statistically significant results, with mean difference BA-CA ranging from -0.20 (at 10 years old) to 0.34 (at 14 years old).

For Asians, five studies were included [24, 28, 38, 51, 53]. The studies did not show any statistically significant results in females, with mean BA-CA ranging from -0.27 (at 6 years old) to 0.50 years (at 15 years old). In males, however, the studies showed statistically significant results for the following ages:

- Six years: overall mean difference BA-CA of -1.08 years (95% CI -1.49, -0.67)
- Seven years: overall mean difference BA-CA of -1.35 years (95% CI -1.85, -0.85)
- Eight years: overall mean difference BA-CA of -1.07 years (95% CI -1.97, -0.17)
- Nine years: overall mean difference BA-CA of -0.80 years (95% CI -1.43, -0.18)
- Seventeen years: overall mean difference BA-CA of 0.50 years (95% CI -0.08, 0.93)

**Fig. 6:**
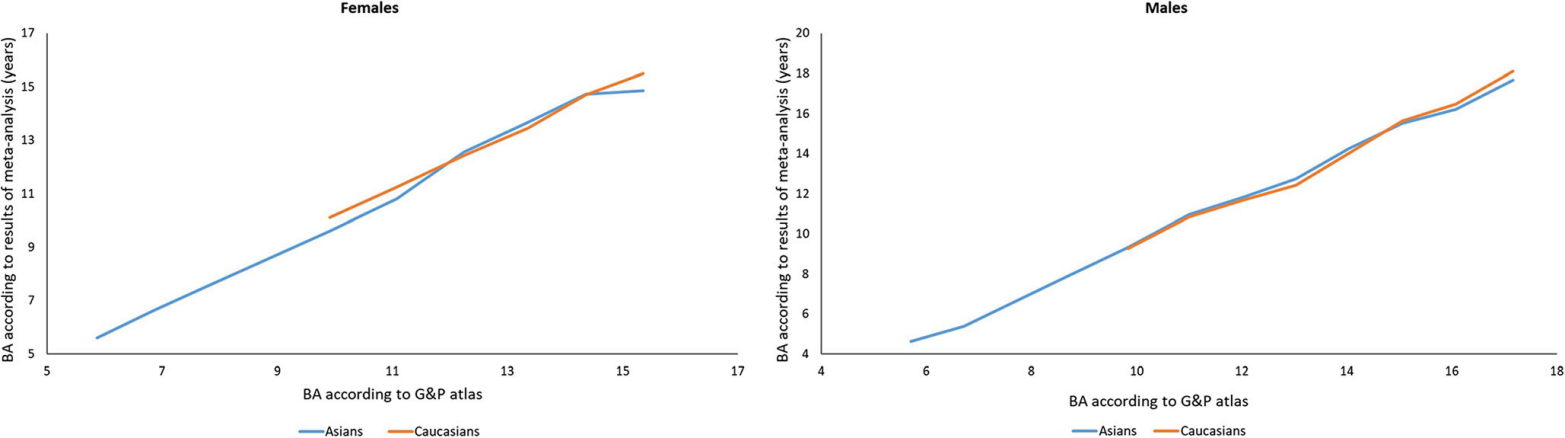
G&P bone age after adjustment based on meta-analysis (females and males)

Based on the results of the yearly interval meta-analysis, we produced graphs for Asians and Caucasians of both sexes (Fig. 6), which show BA according to our meta-analysis compared to BA as assessed by the G&P atlas.

## Discussion

Bone age assessment is a frequently employed and (in the clinical setting) useful diagnostic technique. Its utility in assessing the age of immigrants and asylum seekers is less secure. Figures from the European Commission estimated that in 2016, about 95,000 unaccompanied minors migrated to Europe, of which more than half were Asians [1]. Although there are no exact figures, many of these immigrants were without valid documents to prove their age. Being unable to prove age, or incorrectly assessing a child as an adult, can restrict the child from having access to their rights such as healthcare and education [56] granted by the law in European countries. Hence, it is important that reliable age estimation methods are used.

Concerned with the reliability of the G&P atlas for different ethnic populations, we considered it important to ascertain its applicability to healthy children. Additionally, bias in studies can result in poor reproducibly and/or lead to distorted results and wrong conclusions. However, in this systematic review, results of the four studies with high risk of bias [8, 23, 29, 46] had little impact on (the statistical significance of) our results. This is because the population of these studies contributed less than 5% to the total included population in which only two studies [8, 29] were included in the meta-analysis, which reduced their impact on sample size and results. A funnel plot shows the absence of a large study with high power as most of the studies scattered toward the bottom; however, minimal risk of publication bias was observed among the studies with three studies switched from the funnel plot (Supplementary Fig. 6) [47, 48, 52].

The G&P atlas appears to be applicable to Caucasians, although some recent studies (included in the meta-analysis) have reported that bone age is advanced compared to chronological age in girls up to 13 years old and in boys aged 10 years and above, possibly highlighting the fact that children nowadays are maturing faster than when the atlas was established [32, 42]. Calfee et al [32] assessed the bone age of predominately Caucasian American adolescents (where the G&P atlas was developed). Their skeletal maturation exceeded their chronological age indicating advanced bone age. Perhaps this should not be surprising as Himes [57] reported that skeletal maturation increases by about 0.22 to 0.66 years per decade.

This systematic review and meta-analysis showed no significant difference between BA and CA in Caucasians, which indicates that the G&P atlas is applicable to this group. This is in line with an earlier meta-analysis conducted by Serinelli et al [58] in which no significant difference between BA and CA were found. Note that Serinelli et al included a smaller number of studies; only reported the overall mean difference between BA and CA and did not account for individual age groups.

Concerning the Asian population, three studies recruited Asians living in America [17, 31, 47] while the remaining 17 studies were all carried out in Asia. It seems that skeletal maturation does not conform to the G&P standard at least for some of those who live in East and South Asia. In boys, delay in skeletal maturity during early and middle childhood was followed by advancement during adolescence. Our meta-analysis confirms that there are significant differences between BA and CA in Asian males in two age categories: those aged 6 to 9 years and those aged 17 years. These differences are larger than the standard deviations reported in the G&P atlas for the corresponding age group (± 0.77, ± 0.84, ± 0.90 years at age of 6, 7, 8, and 9 years, respectively), which may have an impact on patient diagnosis and management. In the clinical context, a healthy Asian boy in early childhood could be misdiagnosed as having delayed bone age when using the G&P atlas. The significant advancement in BA compared to CA in Asian males at age 17 is important because this is a critical age in the forensic/legal context, with the individual judged by adult standards in certain legal instances [59].

The G&P standard also seems to be imprecise for Africans. Our meta-analysis of three papers [17, 20, 47] showed significant advancement in bone age of females at all ages (*p* < 0.01). Results from meta-regression with covariates support this difference with BA in Africans being statistically different (Fig. 3). Although our meta-analysis did not show significant difference between BA and CA in African males, some studies reported significant advancement (*p* < 0.01) in adolescence among African American males [15, 17, 47]. Concerning those living in Africa, some studies have shown retardation of bone age among males and females [23, 25, 36]. It is difficult to attribute these variations between Africans only to differences in socioeconomic status, as they were not reported across all studies.

In contrast, the G&P standard appears appropriate for the Hispanic population until adolescence. Our meta-analysis shows no significant difference between BA and CA although only three studies were included [17, 18, 47]. However, Zhang et al, reported that the G&P significantly overestimated males aged between 10 and 13 years [31].

In the current review, a final analysis was performed combining Asians and Hispanics in order to compare our results to those of Serinelli et al, who used the Cavalli-Sforza classification of ethnicity [60], in which Asians and Hispanics are under one ethnic group (Mongoloid). Our meta-analysis of Asian Hispanics for both females and males showed no significant results (Suppl. Fig.6). This is in contrast to Serinelli’s meta-analysis, in which the G&P atlas significantly overestimated chronological age [58]. However, Serinelli et al included only three papers for the Mongoloid population: one related to the Asian population and two to the Hispanic population. One of these latter two studies [61] was excluded from the current systematic review because it included unhealthy children. We therefore believe our results to be more robust.

The major limitation identified in this review is the difficulty in separating ethnicity from socioeconomic status. Relatively few studies reported the socioeconomic status of their sample [9–12, 20, 22, 26, 27, 30, 31, 38, 42, 46, 48, 51]. Children in these studies seemed to follow the same pattern of advancement and delay in bone age as their peers of the same ethnicity in other studies. When bone age is accelerated, new social and cultural factors rather than economic conditions have been suggested to be the main drive [27]. However, our results suggest ethnicity should also be considered when assessing bone age. A further limitation of the study is the failure to calculate the mean absolute and root mean square errors, which might have further confirmed the accuracy of the G&P atlas in relation to each population. However, the mean of each variable (BA and CA) was only available for 13 studies [18, 19, 24, 26–28, 35, 38, 49–53], and for these 13 studies, individual observations were not provided; therefore, the mean error could not be calculated.

## Conclusion

This systematic review revealed that the ethnicity/origin of the child can influence the applicability of the G&P standard. The G&P standard is imprecise and should be used with caution in Asian and African populations, particularly when assessing age for forensic/legal purposes. Some caution is also required for Hispanics (particularly males). The G&P atlas can be used with most confidence in Caucasians. There is a complex inter-relationship between the impacts of socioeconomic status and ethnicity on bone age using the G&P atlas, which no study has clearly set out to address. Although the graphs in Fig. 6 may be helpful, until new ethnicity-related standards are created, clinicians should be aware of the limitations of the G&P method presented in this review.

## Electronic supplementary material

ESM 1 (DOCX 659 kb)

## Abbreviations

BA: Bone age
CA: Chronological age
EV: External validity
G&P: Greulich and Pyle
IV: Internal validity
NICE: The National Institute for Health and Care Excellence
TW: Tanner and Whitehouse

## References

[1] Menjívara C, Perreirab KM (2017). Undocumented and unaccompanied: children of migration in the European Union and the United States. J Ethn Migr Stud.

[2] Ritz-Timme S, Cattaneo C, Collins MJ (2000). Age estimation: the state of the art in relation to the specific demands of forensic practise. Int J Legal Med.

[3] Greulich W, Pyle I (1959). Radiographic atlas of skeletal development of the hand and wrist.

[4] Tanner JM, Healy MJR, Goldstein H, Cameron N (2001) Assessment of skeletal maturity and prediction of adult height (TW3 method). WB Saunders, London

[5] National Institute for Health and Care Excellence. Methods for the development of NICE public health guidance (third edition) | Guidance and guidelines [Internet]. NICE; 2012 [cited 2017 May 18]. Available from: https://www.nice.org.uk/process/pmg4/chapter/appendix-g-quality-appraisal-checklist-quantitativestudies-reporting-correlations-and 27905711

[6] R Core Team (2015) R: a language and environment for statistical computing. R Foundation for Statistical Computing, Vienna

[7] Demisch A, Wartmann P (1956). Calcification of the mandibular third molar and its relation to skeletal and chronological age in children. Child Dev.

[8] Hansman CF, Maresh MM (1961). A longitudinal study of skeletal maturation. Am J Dis Child.

[9] Johnston FE (1963). Skeletal age and its prediction in Philadelphia children. Hum Biol.

[10] Andersen E (1971). Comparison of Tanner-Whitehouse and Greulich-Pyle methods in a large scale Danish survey. Am J Phys Anthropol.

[11] Roche AF, Davila GH, Eyman SL (1971). A comparison between Greulich-Pyle and Tanner-Whitehouse assessments of skeletal maturity. Radiology.

[12] Wenzel A, Droschl H, Melsen B (1984). Skeletal maturity in Austrian children assessed by the GP and the TW-2 methods. Ann Hum Biol.

[13] So LL, Yen PK (1990). Secular trend in skeletalmaturation in southern Chinese girls in Hong Kong. Z Morphol Anthropol.

[14] So LL (1991) Correlation of skeletal maturation with stature and body weight of Southern Chinese girls in Hong Kong. Z Morphol Anthropol 78(3):307–312 Available from: http://www.jstor.org/stable/25757319 1887660

[15] Loder RT, Estle DT, Morrison K et al (1993) Applicability of the Greulich and Pyle skeletal age standards to black and white children of today. Am J Dis Child 147(12):1329–1333 10.1001/archpedi.1993.02160360071022 8249956

[16] Kullman L (1995). Accuracy of two dental and one skeletal age estimation methods in Swedish adolescents. Forensic Sci Int.

[17] Ontell FK, Ivanovic M, Ablin DS, Barlow TW (1996). Bone age in children of diverse ethnicity. Am J Roentgenol.

[18] Jiménez-Castellanos J, Carmona A, Catalina-Herrera C, Viñuales M (1996). Skeletal maturation of wrist and hand ossification centers in normal Spanish boys and girls: a study using the Greulich-Pyle method. Acta Anat (Basel).

[19] Koc A, Karaoglanoglu M, Erdogan M, Kosecik M, Cesur Y (2001). Assessment of bone ages: is the Greulich-Pylemethod sufficient for Turkish boys?. Pediatr Int.

[20] Mora S, Boechat MI, Pietka E, Huang HK, Gilsanz V (2001). Skeletal age determinations in children of European and African descent: applicability of the Greulich and Pyle standards. Pediatr Res.

[21] van Rijn RR, Lequin MH, Robben SG, Hop WC, Van Kuijk C (2001). Is the Greulich and Pyle atlas still valid for Dutch Caucasian children today?. Pediatr Radiol.

[22] Krailassiri S, Anuwongnukroh N, Dechkunakorn S (2002). Relationships between dental calcification stages and skeletal maturity indicators in Thai individuals. Angle Orthod.

[23] Lewis CP, Lavy CB, Harrison WJ (2002) Delay in skeletal maturity in Malawian children. J Bone Joint Surg Br 84(5):732–734 Available from: http://www.bjj.boneandjoint.org.uk/cgi/doi/10.1302/0301-620X.84B5.12642 10.1302/0301-620x.84b5.12642 12188494

[24] Chiang KH, Chou ASB, Yen PS et al (2005) The reliability of using Greulich-Pyle method to determine children’s bone age in Taiwan. Tzu Chi Med J 6:15–18 Available from: http://www.tzuchi.com.tw/tzuchi/tcmj/94-6/1-4.pdf

[25] Garamendi PM, Landa MI, Ballesteros J, Solano MA (2005). Reliability of the methods applied to assess age minority in living subjects around 18 years old: a survey on a Moroccan origin population. Forensic Sci Int.

[26] Haiter-Neto F, Kurita LM, Menezes AV, Casanova MS (2006). Skeletal age assessment: a comparison of 3 methods. Am J Orthod Dentofacial Orthop.

[27] Büken B, Safak AA, Yazici B, Büken E, Mayda AS (2007) Is the assessment of bone age by the Greulich-Pyle method reliable at forensic age estimation for Turkish children? Forensic Sci Int 173(2–3):146–153 Available from: http://pubs.rsna.org/doi/abs/10.1148/radiol.2481071451 10.1016/j.forsciint.2007.02.023 17391883

[28] Griffith JF, Cheng JCY (2007) Are Western skeletal age standards applicable to Hong Kong Chinese? – a comparison of Greulich and Pyle and TW3 methods. Hong Kong Med J 13(3):S28–S32

[29] Schmidt S, Koch B, Schulz R, Reisinger W, Schmeling A (2007). Comparative analysis of the applicability of the skeletal age determination methods of Greulich–Pyle and Thiemann–Nitz for forensic age estimation in living subjects. Int J Legal Med.

[30] Büken B, Erzengin ÖU, Büken E, Şafak AA, Yazici B, Erkol Z (2009). Comparison of the three age estimation methods: which is more reliable for Turkish children?. Forensic Sci Int.

[31] Zhang A, Sayre JW, Vachon L, Liu BJ, Huang HK (2009). Racial differences in growth patterns of children assessed on the basis of bone age. Radiology.

[32] Calfee RP, Sutter M, Steffen JA, Goldfarb CA (2010). Skeletal and chronological ages in American adolescents: current findings in skeletal maturation. J Child Orthop.

[33] Zafar AM, Nadeem N, Husen Y, Ahmad MN (2010). An appraisal of greulich-pyle atlas for skeletal age assessment in Pakistan. J Pak Med Assoc.

[34] Santos C, Ferreira M, Alves FC, Cunha E (2011). Comparative study of Greulich and Pyle atlas and Maturos 4.0 program for age estimation in a Portuguese sample. Forensic Sci Int.

[35] Cantekin K, Celikoglu M, Miloglu O, Dane A, Erdem A (2012). Bone age assessment: the applicability of the Greulich-Pylemethod in Eastern Turkish Children. J Forensic Sci.

[36] Dembetembe KA, Morris AG (2012) Is Greulich-Pyle age estimation applicable for determining maturation in male Africans? S Afr J Sci 108(9–10) Available from: http://www.scielo.org.za/scielo.php?script=sci_arttext&pid=S0038-23532012000500015

[37] Moradi M, Sirous M, Morovatti P (2012). The reliability of skeletal age determination in an Iranian sample using Greulich and Pyle method. Forensic Sci Int.

[38] Patil ST, Parchand MP, Meshram MM, Kamdi NY (2012). Applicability of Greulich and Pyle skeletal age standards to Indian children. Forensic Sci Int.

[39] Santoro V, Roca R, De Donno A (2012). Applicability of Greulich and Pyle and Demirijan aging methods to a sample of Italian population. Forensic Sci Int.

[40] Soudack M, Ben-Shlush A, Jacobson J, Raviv-Zilka L, Eshed I, Hamiel O (2012). Bone age in the 21st century: is Greulich and Pyle’s atlas accurate for Israeli children?. Pediatr Radiol.

[41] Suri S, Prasad C, Tompson B, Lou W (2013). Longitudinal comparison of skeletal age determined by the Greulich and Pyle method and chronologic age in normally growing children, and clinical interpretations for orthodontics. Am J Orthod Dentofacial Orthop.

[42] Hackman L, Black S (2013). The reliability of the Greulich and Pyle atlas when applied to a modern Scottish population. J Forensic Sci.

[43] Paxton ML, Lamont AC, Stillwell AP (2013). The reliability of the Greulich-Pyle method in bone age determination among Australian children: bone age determination using Greulich-Pyle. J Med Imaging Radiat Oncol.

[44] Shilpa PH, Sunil RS, Sapna K, Kumar NC (2013) Estimation and comparison of dental, skeletal and chronologic age in Bangalore south school going children. J Indian Soc Pedod Prev Dent 31(2):63 Available from: http://www.jisppd.com/text.asp?2013/31/2/63/115696 10.4103/0970-4388.115696 23886714

[45] Awais M, Nadeem N, Husen Y, Rehman A, Beg M, Khattak YJ (2014) Comparison between Greulich-Pyle and Girdany-Golden methods for estimating skeletal age of children in Pakistan. J Coll Physicians Surg Pak 24(12):889–893 Available from: https://www.jcpsp.pk/archive/2014/Dec2014/03.pdf 25523722

[46] Rai V, Saha S, Yadav G, Tripathi AM, Grover K (2014) Dental and skeletal maturity- a biological indicator of chronologic age. J Clin Diagn Res 8(9):ZC60–ZC64 10.7860/JCDR/2014/10079.4862 PMC4225977 25386525

[47] Mansourvar M, Ismail MA, Raj RG (2014). The applicability of Greulich and Pyle atlas to assess skeletal age for four ethnic groups. J Forensic Leg Med.

[48] Manzoor Mughal A, Hassan N, Ahmed A (2014) The applicability of the Greulich & Pyle Atlas for bone age assessment in primary schoolgoing children of Karachi, Pakistan. Pak J Med Sci 30(2):409–412 PMC3999020 24772153

[49] Gungor OE, Celikoglu M, Kale B, Gungor AY, Sari Z (2015). The reliability of the Greulich and Pyle atlas when applied to a southern Turkish population. Eur J Dent.

[50] Kim JR, Lee YS, Yu J (2015). Assessment of bone age in prepubertal healthy Korean children: comparison among the Korean standard bone age chart, Greulich-Pyle method, and Tanner-Whitehouse method. Korean J Radiol.

[51] Mohammed RB, Rao DS, Goud AS, Sailaja S, Thetay AA, Gopalakrishnan M (2015) Is Greulich and Pyle standards of skeletal maturation applicable for age estimation in South Indian Andhra children? J Pharm Bioallied Sci 7(3):218–225 Available from: http://www.ncbi.nlm.nih.gov/pubmed/26229357. http://www.pubmedcentral.nih.gov/articlerender.fcgi?artid=PMC4517325 10.4103/0975-7406.160031 PMC4517325 26229357

[52] Öztürk F, Karataş OH, Mutaf HI, Babacan H (2016) Bone age assessment: comparison of children fromtwo different regions with the Greulich–Pyle method in Turkey. Aust J Forensic Sci Taylor & Francis 48(6):694–703. https://www.tandfonline.com/doi/full/10.1080/00450618.2015.1119311

[53] Patel PS, Chaudhary AR, Dudhia BB, Bhatia PV, Soni NC, Jani YV (2015) Accuracy of two dental and one skeletal age estimation methods in 6–16 year old Gujarati children. J Forensic Dent Sci 7(1):18 Available from: https://www.ncbi.nlm.nih.gov/pmc/articles/PMC4330614/10.4103/0975-1475.150298 PMC4330614 25709315

[54] Zabet D, Rérolle C, Pucheux J, Telmon N, Saint-Martin P (2014). Can the Greulich and Pylemethod be used on French contemporary individuals?. Int J Legal Med.

[55] Maggio A, Flavel A, Hart R, Franklin D (2016) Assessment of the accuracy of the Greulich and Pyle hand-wrist atlas for age estimation in a contemporary Australian population. Aust J Forensic Sci Taylor & Francis 618(February):1–11. https://www.tandfonline.com/doi/full/10.1080/00450618.2016.1251970

[56] Feijen L (2008). The Challenges of ensuring protection to unaccompanied and separated children in composite flows in Europe. Refug Surv Q.

[57] Himes JH (1984). An early hand-wrist atlas and its implications for secular change in bone age. Ann Hum Biol.

[58] Serinelli S, Panetta V, Pasqualetti P, Marchetti D (2011). Accuracy of three age determination X-ray methods on the left hand-wrist: a systematic review and meta-analysis. Leg Med (Tokyo).

[59] Cole TJ (2015). The evidential value of developmental age imaging for assessing age of majority. Ann Hum Biol.

[60] Cavalli-Sforza LL, Chisholm B, Menozzi P, Piazza A (1995). The history and geography of human genes. J Asian Stud.

[61] Holderbaum RM, Veeck EB, Oliveira HW, Silva CL, Fernandes A (2005) Comparison among dental, skeletal and chronological development in HIV-positive children: a radiographic study. Braz Oral Res 19(3):209–215 10.1590/s1806-83242005000300010 16308610

