# Supplementary material for: Is the Greulich and Pyle atlas applicable to all ethnicities? A systematic review and meta-analysis
Source: Eur Radiol. 2019 Jan 7;29(6):2910–23. doi: 10.1007/s00330-018-5792-5 (PMC6510872; doi:10.1007/s00330-018-5792-5)
Supplement: Supplementary file 1 — (DOCX 659 kb) [file 330_2018_5792_MOESM1_ESM.docx]

**Supplementary Tables and Figures**

**Supplementary Table1:** Search terms used in each database

| Database | Search Hits* | No. of articles |
| --- | --- | --- |
| PubMed | ((Greulich and Pyle)) OR Greulich Pyle | 223 |
|  | (left hand) AND ((bone age assessment OR bone age determination)) | 247 |
| Cochrane | Age determination by skeleton [MeSH] | 97 |
|  | Greulich and Pyle | 10 |
| Embase | Greulich and Pyle | 340 |

* each row was represent a single search.

**Supplementary table 2**: results of meta-regression with covariates (including sex and ethnicity as explanatory variable)

|  | Estimate | Standard error | Z value | CI% | | P |
| --- | --- | --- | --- | --- | --- | --- |
|  |  |  |  | Lower | upper |  |
| Intercept | -0.01 | 0.01 | -0.18 | -0.21 | 0.17 | 0.85 |
| Sex | 0.06 | 0.12 | 0.52 | -0.17 | 0.30 | 0.59 |
| Asian | 0.01 | 0.14 | 0.08 | -0.26 | 0.28 | 0.93 |
| African | 0.48 | 0.19 | 2.47 | 0.10 | 0.86 | 0.013 |
| Hispanic | 0.02 | 0.22 | 0.11 | -0.42 | 0.47 | 0.90 |

Note: Caucasian are the reference group

**Supplementary Table 3:** Maximum delay and advancement in BA compared to CA using the G&P atlas on a yearly basis as reported in the literature (Caucasian females)

| Age group  (years) | Range of differences  in mean BA-CA (in years) | References |
| --- | --- | --- |
| 7 | -0.40 to 0.20 | (Cantekin et al., 2012, Wenzel et al., 1984) |
| 8 | -0.59 to -0.20 | (Andersen, 1971, Johnston, 1963) |
| 9 | -0.97 to 0.47 | (Andersen, 1971, Hackman and Black, 2013) |
| 10 | -0.47 to 0.40 | (Andersen, 1971, Zabet et al., 2015) |
| 11 | -0.63 to 0.58 | (Andersen, 1971, Bueken et al., 2009) |
| 12 | -0.39 to 0.57 | (Andersen, 1971, Bueken et al., 2009) |
| 13 | -0.19 to 0.75 | (Andersen, 1971, Bueken et al., 2007) |
| 14 | -0.25 to 1.40 | (Andersen, 1971, Zabet et al., 2015) |
| 15 | -0.32 to 1.20 | (Andersen, 1971, Zabet et al., 2015) |
| 16 | 0.95 | (Bueken et al., 2007) |
| 17 | -0.65 to 0.58 | (Hackman and Black, 2013, Johnston, 1963) |
| 18 | -0.90 | (Hackman and Black, 2013) |

*A positive value indicates that the child’s bone age (BA) exceeds his/her chronological age (CA) while a negative value indicates a delay in BA compared to CA

**Supplementary Table 4:** Maximum delay and advancement in BA compared to CA using the G&P atlas on a yearly basis as reported in the literature (Caucasian males)

| Age group (years) | Range of differences  in mean BA-CA (in years)* | References |
| --- | --- | --- |
| 7 | -0.70 to 0.20 | (Cantekin et al., 2012, Wenzel et al., 1984) |
| 8 | -0.85 to 0.15 | (Andersen, 1971, Johnston, 1963) |
| 9 | -0.54 to 0.30 | (Koc et al., 2001, Johnston, 1963) |
| 10 | -0.43 to 0.58 | (Andersen, 1971, Johnston, 1963) |
| 11 | -0.45 to 0.65 | (Andersen, 1971, Johnston, 1963) |
| 12 | -0.27 to 0.59 | (Andersen, 1971, Johnston, 1963) |
| 13 | -0.70 to 0.45 | (Wenzel et al., 1984, Johnston, 1963) |
| 14 | -0.70 to 0.50 | (Wenzel et al., 1984, Zabet et al., 2015) |
| 15 | -1.3 to 1.3 | (Wenzel et al., 1984, Zabet et al., 2015) |
| 16 | -0.66 to 0.98 | (Andersen, 1971, Bueken et al., 2007) |
| 17 | -0.02 to 0.95 | (Bueken et al., 2007, Cantekin et al., 2012) |
| 18 | -0.02 to 0.60 | (Suri et al., 2013, Bueken et al., 2007) |

*A positive value indicates that the child’s bone age (BA) exceeds his/her chronological age (CA) while a negative value indicates a delay in BA compared to CA

**Supplementary Table 5:** Maximum delay and advancement in BA compared to CA using the G&P atlas on a yearly basis as reported in the literature (Asian females)

| Age group  (years) | Average  Mean BA-CA  (range of differences)* | References |
| --- | --- | --- |
| 6 | -0.07 to -0.42 | (Chiang et al 2005, Patil et al 2015) |
| 7 | -0.47 to 0.22 | (Chiang et al 2005, Griffith et al 2007) |
| 8 | -0.84 to 0.11 | (Chiang et al 2005, Chiang et al 2005) |
| 9 | -0.60 to 0.52 | (Patil et al., 2012, Mohammed et al., 2015) |
| 10 | -1 to 0.23 | (Mohammed et al., 2015, Kim et al., 2015) |
| 11 | -0.79 to 0 | (Kim et al., 2015, Patel et al., 2015) |
| 12 | -0.87 to 0.22 | (Patil et al., 2012, Patel et al., 2015) |
| 13 | -0.7 | (Mohammed et al., 2015) |
| 14 | -0.51 | (Mohammed et al., 2015) |
| 15 | -1.21 | (Mohammed et al., 2015) |
| 16 | -0.50 to 0.29 | (Patel et al., 2015, Mohammed et al., 2015) |
| 17 | -0.01 to 0.51 | (Griffith et al 2007, Mohammed et al 2015) |

*A positive value indicates that the child’s bone age (BA) exceeds his/her chronological age (CA) while a negative value indicates a delay in BA compared to CA

**Supplementary Table 6:** Maximum delay and advancement in BA compared to CA using the G&P atlas on a yearly basis as reported in the literature (Asian males)

| Age group  (years) | Average  Mean BA-CA  (range of differences)* | Reference |
| --- | --- | --- |
| 6 | -1.47 to -1 | (Chiang et al 2005, Patel et al 2012) |
| 7 | -1.9 to -0.9 | (Patil et al 2012, Griffith et al 2007) |
| 8 | -2.11 to -0.27 | (Patil et al 2012, Patel at al 2015) |
| 9 | -1.71 to 0.32 | (Patil et al., 2012, Mohammed et al., 2015) |
| 10 | -1.11 | (Patil et al., 2012) |
| 11 | -1.11 | (Patil et al., 2012) |
| 12 | -1.46 to 0.12 | (Patil et al., 2012) |
| 13 | -1.39 to 0.45 | (Patel et al., 2015) |
| 14 | -1.75 to 0.19 | (Patel et al., 2015) |
| 15 | -1.08 to 0.58 | (Patel et al., 2015) |
| 16 | -0.68 to 1.21 | (Patil et al., 2012, Mohammed et al., 2015) |
| 17 | 0.22 to 0.82 | (Chaing et al, 2005, Griffiths et al., 2007) |

*A positive value indicates that the child’s bone age (BA) exceeds his/her chronological age (CA) while a negative value indicates a delay in BA compared to CA


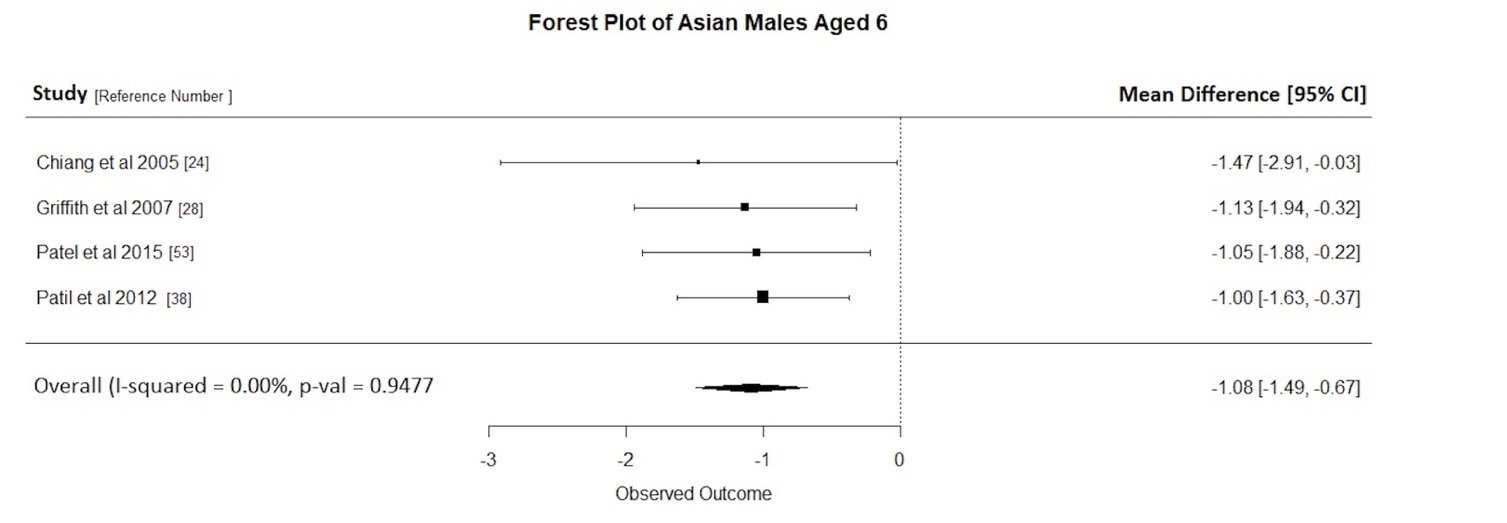
**Supplementary Figure 1:** Forest plot of Asian males (6 years old)


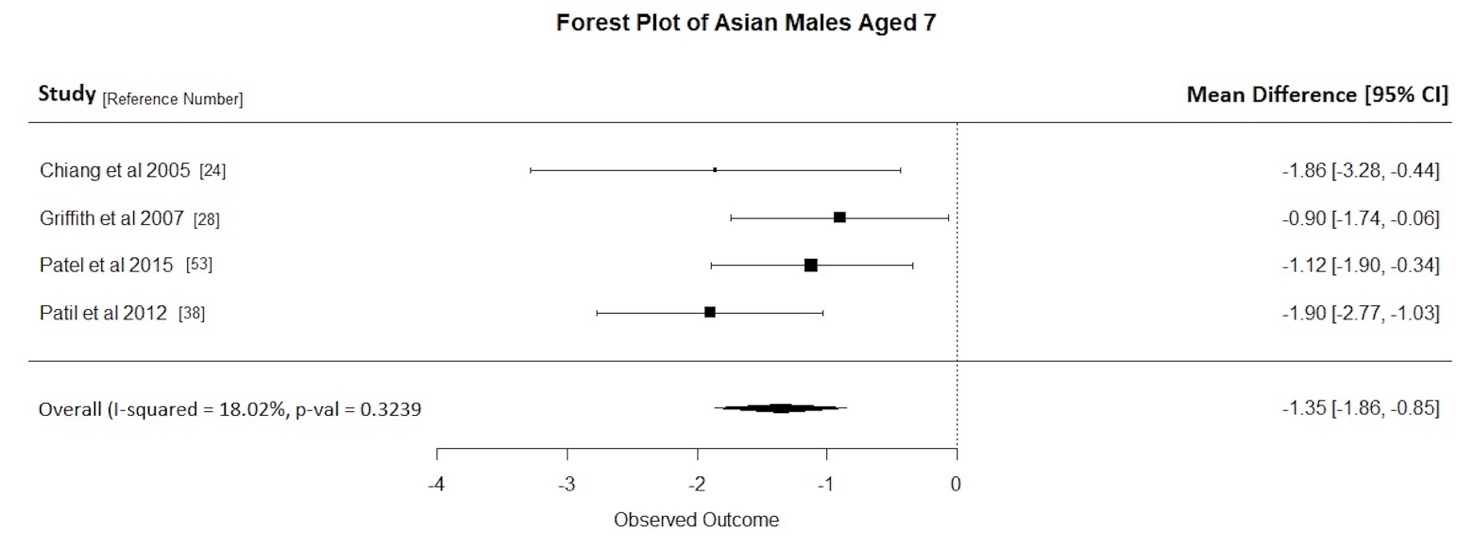
**Supplementary Figure 2:** Forest plot of Asian males (7 years old)


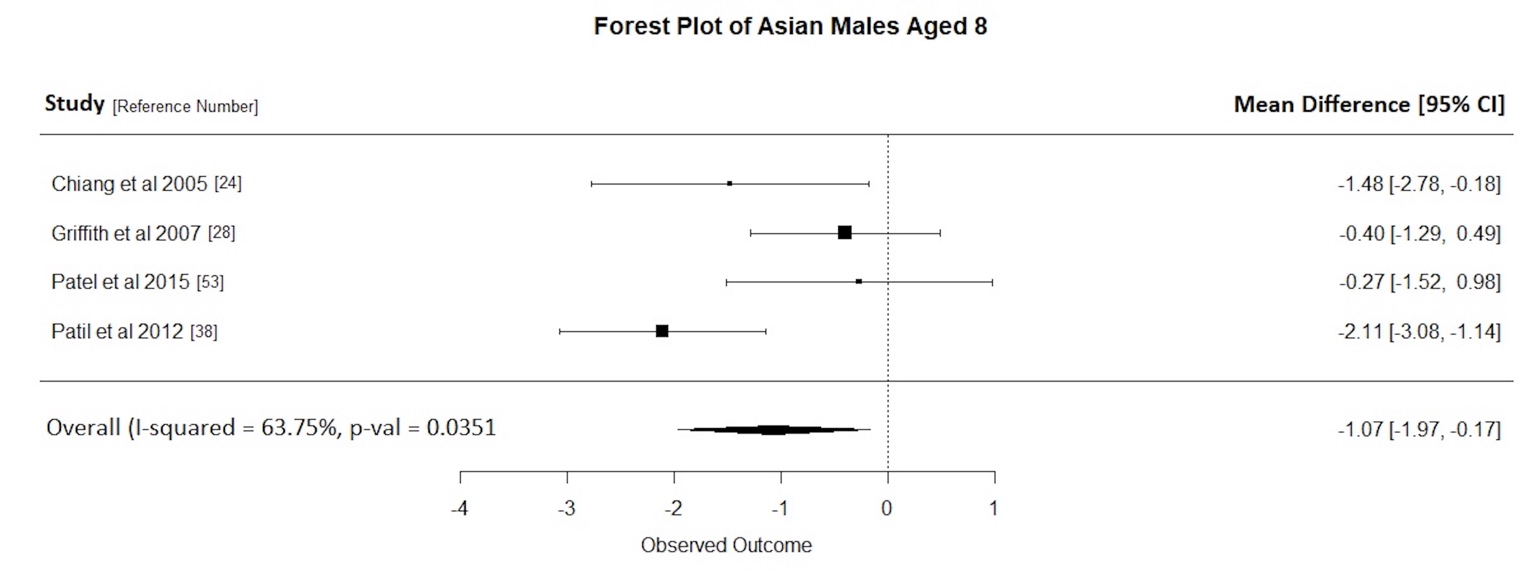
**Supplementary Figure 3:** Forest plot of Asian males (8 years old)


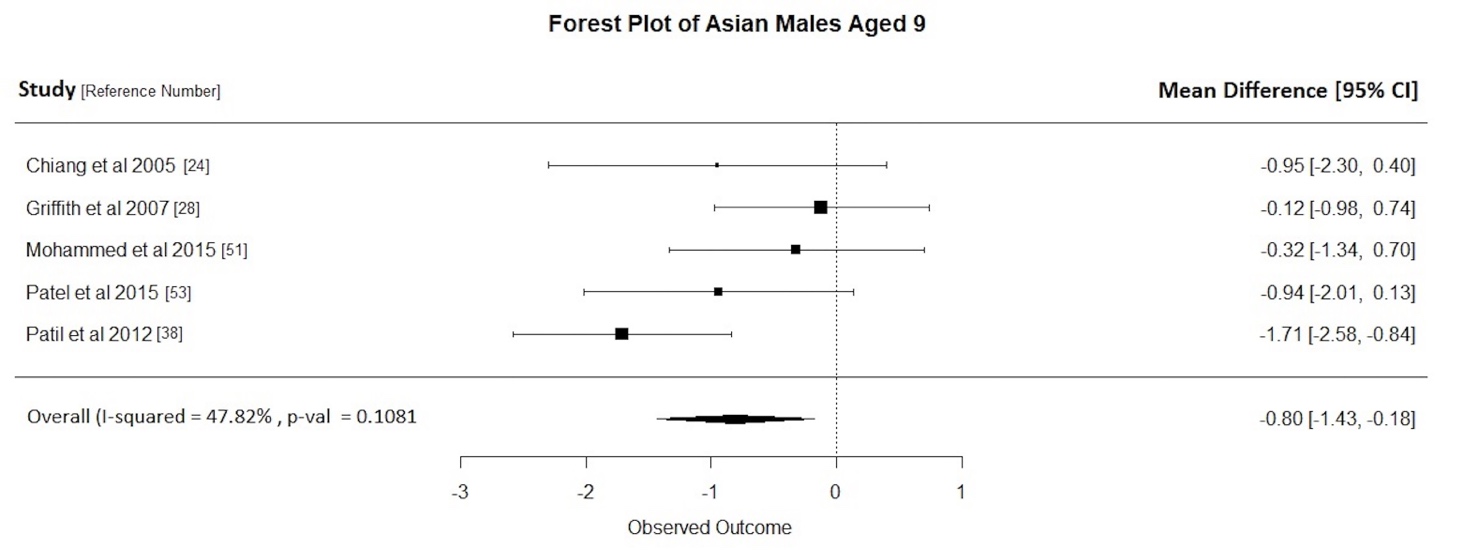
**Supplementary Figure 4:** Forest plot of Asian males (9 years old)


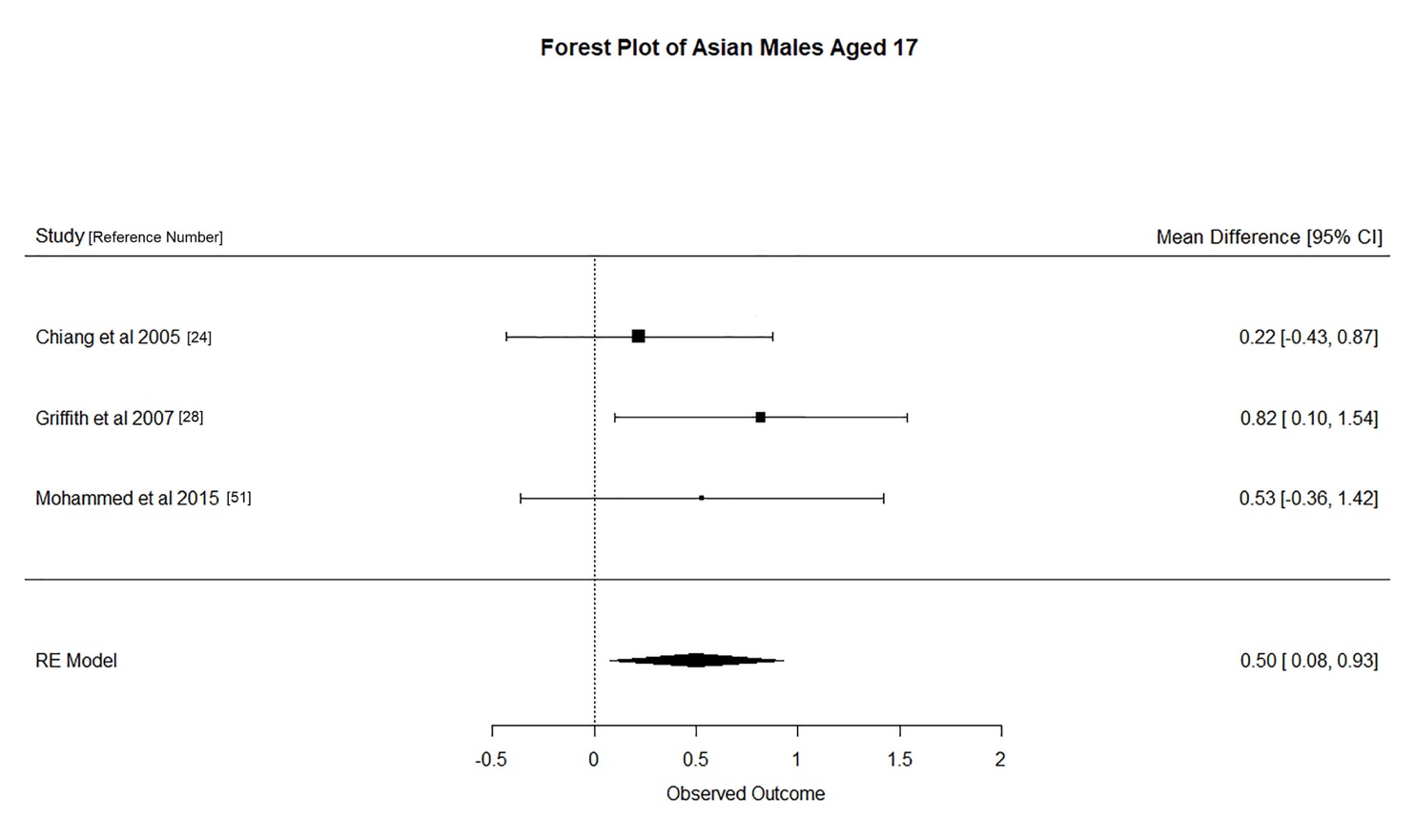
**Supplementary Figure 5:** Forest plot of Asian males (17 years old)


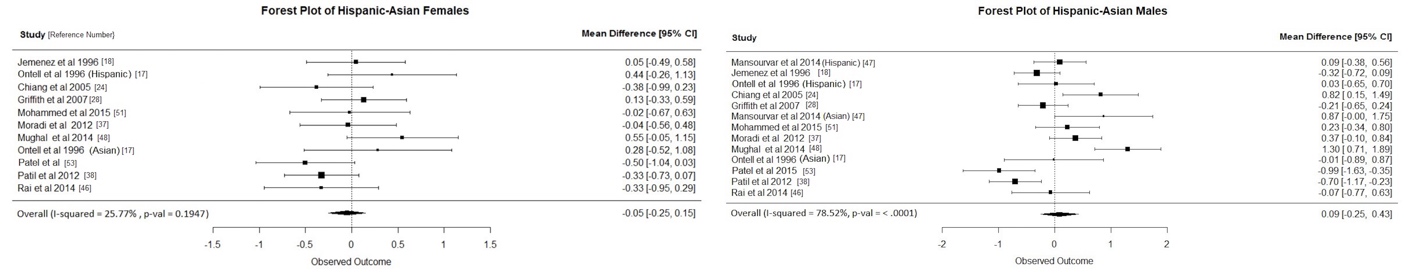
**Supplementary Figure 6:** Funnel plot of standard error plotted against residual value. Large studies with higher power are placed toward the top, while lower powered studies are placed toward the bottom.
